# Effects of Community Environments in Loneliness and Depression among Older Adults in South Korea

**DOI:** 10.1093/geroni/igaf122.2781

**Published:** 2025-12-31

**Authors:** Michin Hong, Soondool Chung

**Affiliations:** Indiana University, Indianapolis, Indiana, United States; Ewha Womans University, Seoul, Republic of Korea

## Abstract

Given its significant impact on older adults’ wellbeing, community environments have gained increasing attention in gerontology. This study examined how community environments affect loneliness and depression among older adults in South Korea and explored their moderating role between the loneliness and depression relationship. We used data from a nationwide survey, focusing on adults aged 65 or older (N = 605). Community environments were measured through service accessibility (SA) and opportunities for community engagement (CE), along with predictors including gender, age, education, perceived health, family network, friend network, and social support. Loneliness and interaction terms for SA, CE, and loneliness were added to the depression model. All scales demonstrated high reliability. Hierarchical regressions were conducted using SPSS 29. Results showed that a greater family network, friend network, social support, SA and CE were associated with lower loneliness. Higher income, better health, a greater family network and social support, lower loneliness, and greater SA were associated with lower depression. Additionally, a moderating effect of SA on the loneliness-depression relationship was found; while living in lower-SV areas was generally associated with higher depression, the negative effect of loneliness on depression was much stronger in individuals living lower-SV areas compared to those in greater-SV areas. The results underscore the critical role of community environments in alleviating loneliness and depression among older adults. Enhancing SA and CE can mitigate loneliness and its harmful effects on depression. Community-based interventions need to be actively implemented to support older adults’ wellbeing.

